# Type 2 diabetes: a sacrifice program handling energy surplus

**DOI:** 10.1093/lifemeta/loae033

**Published:** 2024-08-07

**Authors:** Jianping Ye, Jun Yin

**Affiliations:** Metabolic Disease Research Center, Zhengzhou Key Laboratory of Obesity Research, Zhengzhou Central Hospital Affiliated to Zhengzhou University, Zhengzhou, Henan 450007, China; Tianjian Laboratory of Advanced Biomedical Sciences, Academy of Medical Sciences, Zhengzhou University, Zhengzhou, Henan 450052, China; Department of Endocrinology and Metabolism, Shanghai Sixth People’s Hospital Affiliated to Shanghai Jiao Tong University School of Medicine, Shanghai Clinical Center for Diabetes, Shanghai Diabetes Institute, Shanghai Key Laboratory of Diabetes Mellitus, Shanghai 200233, China

**Keywords:** T2DM, insulin resistance, energy metabolism, ATP, mitochondrial overheating

## Abstract

Type 2 diabetes mellitus (T2DM) is closely associated with obesity, while interactions between the two diseases remain to be fully elucidated. To this point, we offer this perspective to introduce a set of new insights into the interpretation of T2DM spanning the etiology, pathogenesis, and treatment approaches. These include a definition of T2DM as an energy surplus-induced diabetes characterized by the gradual decline of β cell insulin secretion function, which ultimately aims to prevent the onset of severe obesity through mechanisms of weight loss. The body employs three adaptive strategies in response to energy surplus: the first one is adipose tissue expansion to store the energy for weight gain under normal weight conditions; the second one is insulin resistance to slow down adipose tissue expansion and weight gain under overweight conditions; and the third one is the onset of T2DM following β cell failure to reverse the weight gain in obese conditions. The primary signaling molecules driving the compensatory responses are adenosine derivatives, such as adenosine triphosphate (ATP), acetyl coenzyme A (acetyl-CoA), and reduced nicotinamide adenine dinucleotide (NADH). These molecules exert their effects through allosteric, post-translational, and transcriptional regulation of metabolic pathways. The insights suggest that insulin resistance and T2DM are protective mechanisms in the defense against excessive adiposity to avert severe obesity. The perspective provides a unified framework explaining the interactions between the two diseases and opens new avenues in the study of T2DM.

Type 2 diabetes mellitus (T2DM) is one of more than 30 chronic diseases associated with obesity [1]. T2DM care has experienced a quick advance in the past 20 years with the applications of new therapies to clinical practice, while the theoretical question “What exactly is T2DM?” remains unresolved. In a recent international consensus published in *Nature Medicine*, T2DM is defined as “a disease characterized by the gradual decline of β cell insulin secretion function, often accompanied by excessive obesity and insulin resistance” [2]. The definition gives a clinical description of T2DM character. However, the “root” and “biological significance” of T2DM are missing in the definition. The status suggests that the theory about T2DM is left far behind by the clinical practice. This perspective is prepared to address the issue with a set of new viewpoints to interpret the facts on T2DM from an energy-balance angle to address the questions in the etiology, pathogenesis, and treatment. The interpretations are based on principles of feedforward and feedback regulation of energy metabolism in the context of physiology and biochemistry.

## T2DM roots from “energy surplus”

**Figure 1:**
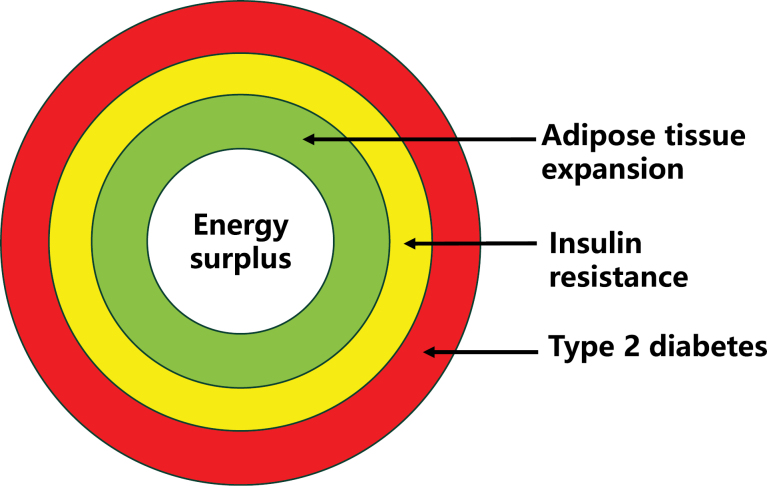
Three defense lines on energy surplus in the maintenance of body weight. There are three defense lines against energy surplus. The first one (green circle) is an expansion of adipose tissue to store excessive energy in the prevention of metabolic disorders. The disorders happen when the adipose compensation reaches the limit in normal subjects. In the subjects with lipodystrophy, the disorders happen in the absence of significant changes in adipose tissue expansion or weight gain. The second one (yellow circle) is insulin resistance to restrain adipose tissue expansion to slow down energy storage and weight gain, which protects insulin-responsive cells to reduce the stress responses from energy oversupply. The third one (red circle) is T2DM to handle excessive energy through urine glucose discharge to prevent excessive adipose tissue accumulation and morbid obesity, which is triggered by β cell failure after long-term compensation to the energy surplus.

It is generally believed that T2DM is a multifactorial disease, depending on factors including genetics, lifestyle, obesity, age, gut microbiota, etc. [3–5]. The “primary cause” of T2DM remains to be established among those factors, although obesity, a state of energy surplus, is widely considered a major factor. Obesity has been extensively studied in the pathogenesis of T2DM, but a consensus remains missing in terms of T2DM theory [3, 4]. This is reflected in the elusive definition of the disease in the consensus and guidelines published recently [2, 6]. According to the principles of physiology and observations by this and other labs, we propose to redefine the disease as follows: T2DM is an energy surplus-induced diabetes characterized by the gradual decline of β cell function to prevent severe obesity by induction of weight loss. The definition is based on a couple of viewpoints about the body’s compensatory response to energy surplus as follows (Fig. 1).

### Adipose tissue expansion

Under normal weight conditions (body mass index [BMI] < 25 kg/m^2^), the adipose tissues undergo expansion in response to energy surplus to store excess energy (triglycerides) leading to weight gain, in which insulin activity is essential for the triglyceride storage in adipocytes. In the postprandial state, energy substances in foods (such as glucose, fatty acids, and amino acids) are absorbed in the intestine and transported to the liver by the portal vein and lymphatic vessels through chylomicrons and very low-density lipoproteins (VLDL). Glucose raises blood insulin levels by triggering insulin secretion from the pancreatic β cells, which is enhanced by fatty acids and amino acids through direct and indirect effects on β cells (such as the glucagon-like peptide-1 [GLP-1] and the gastric inhibitory polypeptide [GIP]) [7]. In response to the elevated glucose and insulin levels, the liver turns on anabolism in the energy storage process to convert glucose into hepatic glycogen for local energy storage and into triglycerides and cholesterol for lipoprotein production in the energy export process. The long-chain fatty acids (LCFAs) are converted into triglycerides, and amino acids are used in the production of proteins. These reactions are involved in the hepatic production of VLDL in the delivery of triglycerides to the peripheral tissues (such as adipose tissue and muscle) [8].

Insulin activity is required for adipose tissue expansion during weight gain, in which insulin promotes the uptake and storage of triglycerides from VLDL in addition to the stimulation of *de novo* lipogenesis in adipocytes. Insulin activates its receptor signaling pathway to change the phosphorylation status of over 2000 proteins in the target cells with 2/3 phosphorylated and 1/3 dephosphorylated [9]. A new study suggests that LCFAs of triglyceride in adipose tissue mainly come from the liver through VLDL or the small intestine through chylomicrons, as excessive biosynthesis of LCFAs generates cytotoxicity in adipocytes, which was observed *in vitro* and *in vivo* by overexpression of fatty acid synthase [10]. There is a competition between glycogen synthesis and fatty acid synthesis in hepatocytes with glycogen priority, in which a new mechanism has been reported recently [11]. Insulin is a primary driving force in the energy storage system for activation of multiple anabolism pathways for glycogen, fatty acids, amino acids, cholesterol, triglycerides, etc. This activity is reduced by insulin resistance in favor of energy mobilization and expenditure. In skeletal muscles, insulin stimulates glucose uptake and glycogen synthesis. However, the insulin activity is not required for muscle glucose uptake as suggested by the phenotype of muscle-specific insulin receptor knockout mice.

### Insulin resistance

Under overweight conditions (BMI > 25 kg/m^2^), the body develops insulin resistance to control excessive weight gain, which decelerates adipose tissue expansion and weight gain. When adipose tissue expansion leads to overweight, adipose tissue dysfunction may slow down the expansion [12]. Insulin resistance is one of the parameters of adipose dysfunction among others, including disorders in lipid storage, adipokine secretion, and cell senescence [12]. Adipocytes exhibit a reduction in glucose uptake, lipogenesis, and triglyceride storage under insulin resistance, which represents a mechanism for the slowdown of adipocyte hypertrophy and adipose tissue expansion. Insulin resistance is a consequence of adipocyte hypertrophy from over storage of triglyceride as found in the system-level analysis of insulin action in multiple mouse strains [13]. Large adipocytes exhibit less insulin sensitivity in both humans and rodents [14]. In mechanism, dysfunction of glucose transporter 4 (GLUT4) is a major event in the insulin resistance of hypertrophic adipocytes [15]. The GLUT4 defect involves at least two mechanisms, such as transcriptional suppression from the carbohydrate response element binding protein (ChREBP) defect and lack of post-translational phosphorylation by adenosine monophosphate (AMP)-activated protein kinase (AMPK) [15]. ChREBP is a transcription factor activated by glucose and fructose to promote the transcription of metabolic genes for *de novo* lipogenesis and triglyceride synthesis in cells including adipocytes [15] and hepatocytes [16]. In addition to carbohydrates, ChREBP also senses reduced nicotinamide adenine dinucleotide (NADH) through an elevated NADH/NAD^+^ ratio (under reductive stress) to promote lipogenesis [17]. Disorders of ChREBP and AMPK contribute to insulin resistance in hypertrophic adipocytes. However, the causes of the disorders remain to be established [15, 18].

Adenosine triphosphate (ATP) surplus provides a potential mechanism for the disorders. To this point, we propose that metabolites of nucleotides (such as ATP, acetyl coenzyme A [acetyl-CoA], NADH) may be the upstream signals for the disorders of AMPK and ChREBP [9, 19]. ATP surplus induces insulin resistance through several mechanisms, including AMPK inhibition, reactive oxygen species (ROS) production, mitochondrial dysfunction, the mechanistic target of rapamycin (mTOR) activation, and the induction of hyperinsulinemia and hyperglucagonemia as reviewed [19, 20]. ATP elevation was reported in the epididymal fat tissue of obese mice fed on a high fat diet [21]. The cause of ATP elevation remains unknown in the adipocytes, but a reduction in ATP consumption by triglyceride biosynthesis is a reasonable factor. Triglyceride biosynthesis is an ATP consumption process as the formation of one molecule of triglyceride from glycerol and fatty acids requires seven molecules of ATP [22]. Triglyceride biosynthesis is reduced in hypertrophic adipocytes, and the mechanisms include lipotoxicity [23], adipose tissue hypoxia [24], and adipocyte senescence [25]. Hyperinsulinemia contributes to adipocyte insulin resistance through the activation of the negative feedback loop of the insulin signaling pathway [26, 27].

Adipocyte insulin resistance contributes to systemic insulin resistance in a couple of ways. Releasing fatty acids for ectopic fat deposition is one of them. Additionally, alteration in the endocrine activities of adipocytes is another mechanism. The adipocytes exhibit an endocrine disorder including secretion of more leptin and inflammatory cytokines with less adiponectin [28]. Leptin, a pro-inflammatory cytokine, acts on the brain to reduce appetite for less energy intake in the control of energy balance [29, 30]. Inflammatory cytokines act on the adipocytes and peripheral tissues to induce lipolysis for energy mobilization [31] and energy expenditure [32]. The adipocyte dysfunctions are aggravated by the adipose tissue hypoxia [33], which is a result of insufficient blood supply [34] together with an increased demand for oxygen [35]. This line of studies suggests that hypertrophic adipocytes contribute to systemic insulin resistance through the disorders in storage and endocrine functions.

ATP surplus in the pancreatic β cells may contribute to systemic insulin resistance through hyperinsulinemia. The systemic insulin resistance is coupled with hyperinsulinemia, a combined result of excessive insulin secretion by the β cells [36, 37] and reduced insulin clearance by the liver [38, 39]. The β cell compensation is generally believed to be a consequence of adaptation to insulin resistance with β cell proliferation and pancreatic islet expansion [36, 37]. However, the molecular mechanism for the compensation remains unclear although several hypotheses have been proposed, including the activation of G-protein-coupled receptors for GLP-1, GIP, and glucagon [40]. Insulin secretion of β cells is stimulated by intracellular ATP derived from energy substrates, including glucose, amino acids, and fatty acids [40]. Excessive ATP is required for the over secretion of insulin by β cells in obese conditions [20]. The excessive supply of glucose, amino acids, and fatty acids provides a perfect basis for the β cell response [20, 40]. The β cells exhibit nutrition sensitivity in the order of glucose > amino acids > fatty acids. However, they are more sensitive to fatty acids than glucose in certain conditions as suggested by a recent proteomics study [41]. Hyperinsulinemia may aggravate systemic insulin resistance through activation of the negative feedback loop of the insulin receptor signaling pathway in insulin target cells, which involves suppression of insulin receptor substrate (IRS) activity by serine kinases including protein kinase B (Akt)/mTOR/S6 kinase (S6K) [9, 27, 42] and inhibition of GLUT4 [9]. This group of studies suggests that energy surplus in the β cells deteriorates systemic insulin resistance through hyperinsulinemia.

ATP surplus in the pancreatic α cells may contribute to systemic insulin resistance through hyperglucagonemia [19, 20]. Over secretion of glucagon by the pancreatic α cells for hyperglucagonemia is a major factor in the pathogenesis of T2DM as documented [43–45]. Glucagon secretion is controlled by several factors, such as amino acids, insulin, glucose, paracrine hormones, and the central nervous system [45]. The disorder of these factors together with elevated α cell mass is the basis for the superactivity of α cells in T2DM. However, the molecular basis of the α cell alteration remains to be established. Intracellular ATP is a physiological signal for glucagon secretion in α cells, and glucagon overproduction is a consequence of ATP supply [20]. Therefore, ATP oversupply in α cells is a reasonable mechanism for hyperglucagonemia. Glucagon contributes to systemic insulin resistance by antagonizing insulin action in multiple tissues, including the liver, adipose tissue, and skeletal muscle [46]. In the liver, glucagon stimulates gluconeogenesis and glycogenolysis for glucose output in the maintenance of blood glucose; in the adipose tissue, glucagon induces lipolysis for fatty acid release in the energy mobilization process; in the skeletal muscle, glucagon induces protein degradation to release amino acids in support of hepatic gluconeogenesis, which happens together with inhibition of glucose uptake in the muscle to spare glucose for the brain. These activities of glucagon work together to prevent hypoglycemia in fasting conditions. However, the activities promote energy mobilization from the adipose tissues and skeletal muscles to enhance energy expenditure. This effect happens through glucagon inhibition of insulin sensitivity in the peripheral tissues, which may be considered a compensative mechanism in the control of energy surplus in obesity. Unfortunately, this activity of α cells contributes to the glucose disorder in T2DM.

Insulin resistance deaccelerates adipose tissue expansion in favor of weight control. Traditionally, insulin resistance is considered to be detrimental for its impact on the pathogenesis of T2DM. However, we propose that insulin resistance is a beneficial response to energy overcharge for the prevention of excessive weight gain [47]. The view may explain insulin resistance in patients without obesity. In lipodystrophy conditions where the adipocytes and white adipose tissue lack storage functions [48], insulin resistance occurs in the absence of fat tissue expansion and weight gain due to a deficiency in adipose plasticity [48, 49]. The lipodystrophy impairs insulin sensitivity by ectopic fat deposition, such as hyperlipidemia and fatty liver, often associated with T2DM [50]. This leads to oversupply of the energy substrates to insulin-sensitive cells in the absence of obesity, leading to mitochondrial overload and excessive ATP production. In normal conditions, ectopic fat deposition happens after a certain degree of adipose tissue expansion, such as overweight and obesity. Therefore, energy surplus provides an explanation for insulin resistance in both obese and non-obese patients.

## T2DM

After long-term obesity (BMI > 28/30 kg/m^2^), energy surplus triggers T2DM to dispose the excess energy (glucose) in urine, resulting in weight loss. Energy surplus causes adaptive responses in multi-organs (such as the pancreas, liver, skeletal muscle, adipose tissue, heart, and brain) as reviewed [8, 50]. Among these, pancreatic β cell failure in compensation is the primary cause of hyperglycemia in T2DM. In the early stage of insulin resistance, energy surplus leads to β cell super-secretion of insulin in response to the excessive glucose, which acts through mitochondrial production of excess ATP in the cells under mitochondrial overheating [20, 47]. This status eventually leads to β cell failure with a reduction in cell number and islet mass. There are multiple hypotheses for the β cell failure. Mitophagy defect is one of them as mitophagy is required for the maintenance of the insulin secretion function of β cells [51]. The deficiency has been reported in T2DM conditions due to lysosomal defects, in which autophagosome-lysosome fusion could not complete from lysosomal inactivation due to reduced expression of lysosomal genes and decreased lysosomal acidification [51]. Treatment of β cells with glucose and fatty acids leads to the lysosomal defect and mitophagy interruption. However, the signaling molecule that mediates the activities of glucose and fatty acids remains unknown [51]. We propose that ATP is the signaling molecule, whose oversupply is known to inhibit mitophagy through AMPK and mTOR [51].

T2DM may preserve lifespan through the prevention of severe obesity. T2DM is usually considered to be detrimental for diabetic complications. However, in terms of the impact on mortality, the diabetic complications are less harmful than cardiovascular failure from severe obesity. Obesity (BMI = 30−39.9) increases the incidence of cardiovascular failure by 50% with a high risk of premature death [52]. With severe obesity (BMI > 37), the lifespan is further reduced by all-cause death. Sumo wrestlers have a short lifespan around 45 years on average from severe obesity [53]. The weight loss from T2DM prevents the onset of severe obesity. Hyperglycemia and hyperglycosuria in T2DM result in weight loss in the absence of medical treatment [8]. In this case, T2DM decreases the risk of severe obesity to prevent the early onset of life-threatening heart failure. In terms of mechanism, a high level of insulin is required for the development of severe obesity. The high activity of insulin is a risk factor for all-cause death as demonstrated in the United Kingdom Prospective Diabetes Study (UKPDS) clinical trial for insulin therapy of T2DM [54]. The insulin activity accelerates the aging process through the activation of mTOR and suppression of the class Ⅲ histone deacetylase sirtuin 1 (SIRT1) [55]. Calorie restriction studies demonstrate that energy surplus reduces lifespan in multiple model systems [56], which is another support for insulin promotion of premature death. In this context, we propose that T2DM may represent a feedback mechanism in response to energy surplus to preserve lifespan.

### Signaling molecules of energy surplus

It is well accepted that in obesity, energy surplus causes T2DM, but the identity of the energy surplus signal remains elusive. Various models have been employed to explore the signal identity in obesity by this and other labs [8, 20, 50, 57, 58]. The results from our studies of chronic inflammation, gut microbial metabolite, herbal extract berberine, and weight loss surgery consistently suggest that molecules in the energy metabolism pathway of mitochondria are candidates for energy surplus signals [20, 47].

ATP is considered as the primary signaling molecule for energy surplus in our studies. As discussed above, ATP may initiate lipogenesis and adipose tissue expansion for weight gain in the initial response to energy surplus, trigger insulin resistance in the second response to energy surplus, and cause the onset of T2DM in the third response to energy surplus. In biochemistry, mitochondria sense various metabolic substrates during dynamic adaptations to energy demand in cells [57, 59, 60]. Specifically, under energy surplus conditions, a large number of substrates enter mitochondria for the mitochondrial overload, triggering the feedforward metabolic reactions, such as increasing ATP production by oxidative phosphorylation leading to an oversupply of intracellular ATP known as “mitochondrial overheating” [20, 47]. As an indicator of energy charge status, intracellular ATP is under strict control. The increased production will induce more ATP consumption as the cells lack an ATP storage system. In the liver, the ATP charge promotes hepatocytes to synthesize lipids in the production of VLDL for energy export, which is an energy-consumption process for energy discharge. Synthesizing one molecule of LCFA at 18 carbons from glucose requires the consumption of 129 molecules of ATP [61]. Synthesis of one molecule of cholesterol from glucose requires the consumption of 18 molecules of ATP [61]. ATP derivatives (adenosine diphosphate (ADP) and AMP) are indicators of energy deficiency. ATP is a reasonable signal of energy surplus.

There are at least five signaling pathways by which ATP contributes to systemic insulin resistance [20]. Firstly, ATP inhibits glucose metabolism through allosteric inhibition of enzymes in the glycolysis pathway [62], which downregulates glycolysis in the insulin-induced glucose metabolism pathway, contributing to insulin resistance. Secondly, ATP inhibits AMPK activity through the elevation of ATP/AMP ratio, leading to reduced GLUT4 translocation in the insulin-induced glucose uptake pathway [63]. Additionally, AMPK suppression aggravates insulin resistance through mitochondrial dysfunction following deficiency in mitochondrial biogenesis and mitophagy [64]. Thirdly, ATP may activate mTOR to suppress signaling activities of the IRS proteins in the insulin receptor signaling pathway by induction of serine phosphorylation [9, 20]. Fourthly, ATP surplus may induce endocrine disorders such as hyperinsulinemia and hyperglucagonemia as discussed above to impair systemic insulin sensitivity. Fifthly, in addition to the activities inside cells, ATP is secreted by cells into the microenvironment to act outside cells by interactions with specific receptors on the cell surface, which has been reviewed for regulation of energy metabolism [19]. It is expected that there are other mechanisms for ATP activities in insulin resistance, given that ATP is produced in all types of cells and has cell-type-specific effects. The β cell-specific effects include superproduction of insulin, and the α cell-specific effects include glucagon secretion. The activities of ATP provide a unifying mechanism for insulin resistance in the adipocytes, hepatocytes, and myocytes in the pathogenesis of T2DM.

Immune cell-specific effects of ATP represent a potential mechanism for chronic inflammation in obesity. ATP induces an inflammatory response in macrophages as reported [21, 65]. Chronic inflammation (low-grade chronic inflammation, metabolic inflammation, or immunometabolism) was believed to be an important risk factor for obesity-associated systemic insulin resistance [66–68]. However, the view has been challenged by studies from this and other labs [69, 70]. Following is a part of the reasons. Firstly, in the adipose tissue, inflammation is required for healthy tissue expansion [71] and blood supply through induction of angiogenesis [69]. Secondly, reduction of inflammation could not improve insulin sensitivity in T2DM patients. Over the past 30 years, strategies targeting inflammation have all ended up in failure in the clinical trials for the improvement of insulin resistance in the patients [20]. Thirdly, recent studies suggest that inflammatory cytokines are required for the maintenance of energy homeostasis and insulin sensitivity in animals [70–73] and humans [32, 74]. These studies suggest that obesity-associated chronic inflammation is likely a protective response from energy surplus to enhance energy expenditure, in which ATP promotes inflammatory responses through intracellular and extracellular mechanisms [19].

In addition to ATP, acetyl-CoA, an upstream intermediate metabolite in the ATP production pathway, is another candidate for energy surplus signal. Production of ATP from acetyl-CoA is dependent on the tricarboxylic acid (TCA) cycle, in which the energy of acetyl-CoA is stored in NADH and flavin adenine dinucleotide (FADH_2_). Thereafter, the energy is used to produce ATP by the mitochondrial respiratory chain through oxidative phosphorylation. As a major intermediate metabolite in the TCA cycle, acetyl-CoA is derived from the upstream metabolic pathways of glucose, fatty acids, and amino acids. Acetyl-CoA is used in the downstream metabolic pathways for the production of ATP, glucose, fatty acids, amino acids, cholesterol, etc. Acetyl-CoA is also used in protein modification of acetylation. Acetyl-CoA may contribute to insulin resistance in a couple of ways. It inhibits insulin activity by acetylation of Akt/mTOR kinases in the insulin signaling pathway [9]. Acetyl-CoA promotes hepatic gluconeogenesis by allosteric activation of pyruvate carboxylase in the gluconeogenic pathway to promote the conversion of pyruvate into oxaloacetic acid, an intermediate metabolite for gluconeogenesis [75]. This effect weakens insulin activity in the inhibition of gluconeogenesis.

NADH is also a candidate of energy surplus signal along ATP and acetyl-CoA [19]. NADH is an intermediate product between acetyl-CoA and ATP. An elevation in NADH has been linked to T2DM and hyperglycemia in a couple of models [76–78]. There are opposite reports in which a reduction in NADH promotes hepatic glucose production [75], a parameter of insulin resistance. The reason for the dispute remains to be explored. NADH is converted into nicotinamide adenine dinucleotide (NAD^+^) through the production of ATP, reduced nicotinamide adenine dinucleotide phosphate (NADPH), lactic acid, etc. The NADH/NAD^+^ ratio is widely used as an indicator of energy status in the aging field for SIRT1 regulation [79] and a parameter of “reductive stress” in the redox field [80]. An elevation of the NADH/NAD^+^ ratio promotes the production of lactic acid from pyruvate in the study of the Warburg effect in the context of reductive stress [81]. In hepatocytes, an elevation of NADH/NAD^+^ ratio promotes *do novo* lipogenesis by activation of ChREBP [17]. In a time-restricted feeding study, an elevation of NADH/NAD^+^ ratio was found in the livers of mice under restricted feeding, which was associated with a decrease in body temperature and a reduction in SIRT1 activity [82]. When the elevation was eliminated by expression of the water-forming NADH oxidase from *Lactobacillus brevis* (LbNOX) in transgenic mice, the reduction in body temperature was blocked, which was associated with SIRT1 activation. Insulin sensitivity was not reported in the study under NADH/NAD^+^ elevation. However, the study supports that NADH/NAD^+^ elevation promotes liver insulin resistance as the indicator of insulin sensitivity, such as enhanced hepatic gluconeogenesis, is associated with the fasting condition. The impact of NADH/NAD^+^ on metabolism is specific to cell types [83]. NADH may preserve the acetylation status of the signaling molecules through the inhibition of histone deacetylase, such as SIRT1 [9]. Glucose inhibits the signaling molecules by O-linked β-N-acetylglucosamine modification (O-GlcNAcylation) [9].

### T2DM therapy for energy surplus

Given that excessive ATP is the root of problems in obesity, it is important to control the ATP charge in the treatment of T2DM. In this case, correction of mitochondrial overload is a potential cutting point in T2DM care. Does clinical practice support this possibility? Our answer is yes. The energy substrates may be considered as floodwater and mitochondria are considered as reservoirs in the energy flow. Excessive floodwater (energy substrates) in the reservoirs will cause dam breaks. In this situation, mitochondria will open “flood channels” through the export of products (such as ATP, acetyl-CoA, and NADH) for the biosynthesis of different products, which include fatty acids, amino acids, nucleotides, cholesterol, ketone bodies, glucose, etc. Production of these substances requires the consumption of ATP to supply energy, which represents one of the energy disposal channels. As an important organ in the pathogenesis of T2DM [84], the liver can use all of the “flood channels” in handling energy surplus. T2DM is the result of opening the “gluconeogenesis channel” in the liver for the hyperglycemia and the “glucose disposal channel” in the kidneys for high urine glucose.

In clinical practice, GLP-1 receptor (GLP-1R) agonists (such as semaglutide) are effective in the treatment of T2DM. The medicines inhibit food intake by producing satiety through targeting the central nervous system to ease the whole-body energy charge [85], which in turn takes care of mitochondrial overload in cells of various organs. In response, hepatocytes turn down the glucose discharge channel favoring blood glucose control, and β cells secret less insulin to attenuate hyperinsulinemia. Sodium-glucose cotransporter 2 (SGLT2) inhibitors (such as dapagliflozin) open the renal “glucose discharge channel” to reduce energy charge in the bloodstream by urine glucose disposal [86]. Both types of drugs lower blood glucose charge, but their mechanisms of action and long-term effects are different. Therefore, management of whole-body energy charge is an effective strategy for taking care of mitochondrial overload and mitochondrial overheating in the effective care of T2DM. This point is reflected in the weight control strategy that is emphasized in the updated guidelines for T2DM treatment by the American Diabetes Association (ADA) [6].

**Figure 2:**
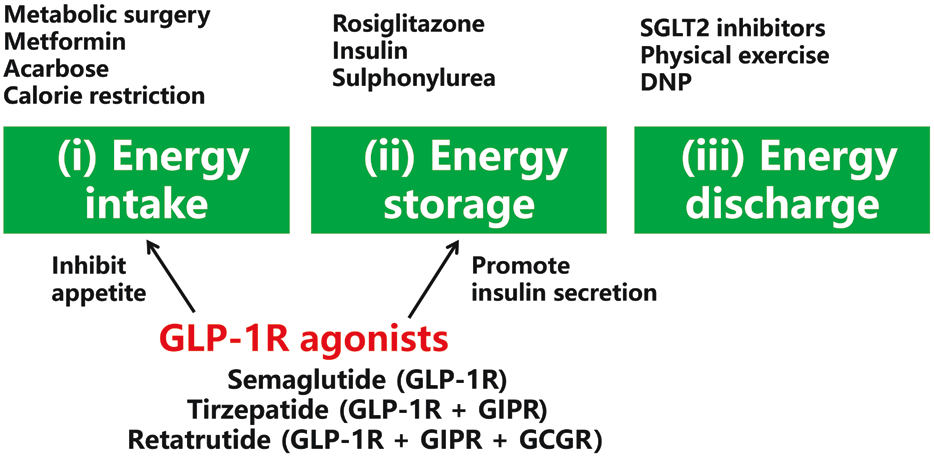
Classification of T2DM care strategies. T2DM care strategies are categorized into three groups according to their impact on energy metabolism. The first group includes metabolic surgery, metformin, acarbose, and calorie restriction, which reduce food intake to attenuate energy surplus. The second group includes rosiglitazone (PPARγ activator), insulin, and sulphonylurea, which promote energy storage through the conversion of glucose into triglycerides. GLP-1R agonists that include single, dual, and triple agonists belong to both groups 1 and 2. The third group includes SGLT2 inhibitors, physical exercise, and uncoupler 2,4-dinitrophenol (DNP), which promote energy discharge.

To support the above views, we classify T2DM care strategies into three categories (Fig. 2). (i) Restricting energy intake: this class of strategies reduces the whole-body energy charge by decreasing food intake, which includes weight loss surgery, metformin [87–89], α-glucosidase inhibitors (such as acarbose), dietary restrictions, etc. (ii) Adjusting energy distribution: this class of strategies reduces blood energy charge by depositing glucose into the peripheral tissues, which includes insulin, sulfonylurea drugs, and peroxisome proliferator-activated receptor γ (PPARγ) activators. (iii) Increasing energy disposal: this class promotes energy disposal to get rid of energy overcharge in the body, which includes SGLT2 inhibitors, physical exercise, heat production, etc. Among these strategies, the metabolic surgery is the only therapy for curing T2DM through effective control of body weight [47]. The class 2 only transfers energy from the blood to the storage tissues, including the adipose tissue, liver, and muscle without solving the total energy surplus in the body. GLP-1R agonists (including semaglutide), dual agonist (tirzepatide), and triagonist (retatrutide) have activities of restricting energy intake and adjusting energy distribution for outstanding efficacy in the treatment of T2DM and obesity care [1, 90]. The dual agonist (GLP-1R and GIP receptor [GIPR] agonist) and triagonist (GLP-1R/GIPR/glucagon receptor agonist) exhibit better activities than the single agonist of GLP-1R in the control of body weight.

In addition, dual agonists of GLP-1R and glucagon receptor exhibit excellent activities in weight control [91, 92]. Activation of glucagon receptor leads to insulin resistance in the liver, fat, and muscle for induction of gluconeogenesis and lipolysis in a way against insulin activity [46]. The increased lipolysis leads to a reduction in fat mass for weight loss, which supports that insulin resistance is a mechanism of weight control in the human body. The view provides a perfect explanation for the recommendation of GLP-1R agonists over insulin in the glycemic control in the updated ADA guideline of T2DM care published in 2024 [93].

## Conclusion

**Figure 3:**
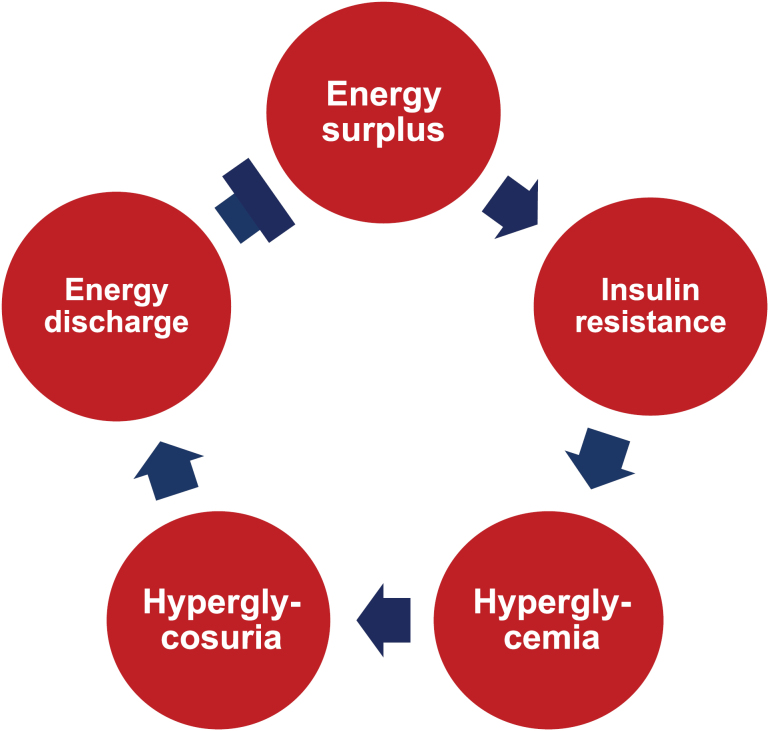
The biological significance of T2DM in obesity. The characteristics of T2DM, including insulin resistance, hyperglycemia, hyperglycosuria, etc., are generated to control energy surplus to prevent excessive adipose tissue expansion and severe obesity.

We present a set of new insights into T2DM to explain the etiology, pathophysiology, and pharmacology. The views are generated by integration of the clinical observations and biochemical and physiological principles, especially the feedforward and feedback regulation of energy metabolism in the conditions of obesity. The viewpoints set up the framework for a unifying mechanism underlying the clinical manifestation and treatment of T2DM, which spans from the whole body, tissue/organ, and cellular levels to the molecular level. The framework explains the facts about obesity in the onset of T2DM and T2DM in the control of obesity in the interaction of two diseases. The energy-based view suggests that T2DM is one of the body’s strategies for handling long-term energy overcharge to reduce the risk of severe obesity (Fig. 3). Insulin resistance is one of the components of T2DM in the self-defense program to assist weight control. This perspective serves as an update on existing hypotheses of insulin resistance, such as lipotoxicity, glucotoxicity, inflammation, mitochondrial dysfunction, hyperinsulinemia, hyperglucagonemia, oxidative stress [20].
